# Butyrate Producers in Very Low Birth Weight Infants with Neither Culture-Proven Sepsis nor Necrotizing Enterocolitis

**DOI:** 10.3390/nu17081329

**Published:** 2025-04-11

**Authors:** Anucha Thatrimontrichai, Manapat Praditaukrit, Gunlawadee Maneenil, Supaporn Dissaneevate, Kamonnut Singkhamanan, Komwit Surachat

**Affiliations:** 1 Division of Neonatology, Department of Pediatrics, Faculty of Medicine, Prince of Songkla University, Songkhla 90110, Thailand; manapatpang@gmail.com (M.P.); kookwadee@hotmail.com (G.M.); dsupapor@medicine.psu.ac.th (S.D.); 2 Department of Biomedical Sciences and Biomedical Engineering, Faculty of Medicine, Prince of Songkla University, Songkhla 90110, Thailand; skamonnu@medicine.psu.ac.th (K.S.); komwit.s@psu.ac.th (K.S.)

**Keywords:** biodiversity, butyrate producers, gut microbiome, microbiota, necrotizing enterocolitis, neonatal sepsis, nutrigenomics, premature infant

## Abstract

**Background/Objectives**: Severe infection (sInfection; either late-onset culture-proven sepsis or necrotizing enterocolitis) in very low birth weight (VLBW) infants increases mortality rates and may show long-term progression. The fecal microbiome composition in VLBW infants with and without sInfection was classified in the sInfection and non-sInfection groups. **Methods**: Gut microbiomes, secondary information from a previous randomized trial, were analyzed using QIIME 2 software. The biodiversity and abundance of the gut microbiota between the sInfection and non-sInfection groups were compared. **Results**: Fifty-one neonates were included in the sInfection (*n* = 9) and non-sInfection (*n* = 42) groups; no significant differences in the fecal microbiome were observed in both alpha and beta diversities. Analysis of relative abundance revealed that in both groups, the predominant gut microbiota phylum, class, and genus were Proteobacteria, Gammaproteobacteria, and *Klebsiella*, respectively. The main fecal microbiome in the non-sInfection group included *Faecalibacterium*, *Clostridium butyricum*, and *Bacteroides fragilis*. *Clostridium_sensu_stricto _1* was significantly more abundant in the non-sInfection group than in the sInfection group. **Conclusions**: *Clostridium_sensu_stricto_1* was the main gut microbiota in the non-sInfection group. Considering the potential taxa as synbiotics (correlations among prebiotics, probiotics, and postbiotics), therapeutics may be useful for preventing and managing necrotizing enterocolitis or late-onset culture-proven sepsis in VLBW infants.

## 1. Introduction

Very low birth weight (VLBW) infants experience health problems associated with microbial exposure, including late-onset culture-proven sepsis (LOS) and necrotizing enterocolitis (NEC), because they have an immature immune system (host defense mechanisms). These infants require multiple medical interventions, such as gastric and endotracheal intubation, central line catheterization, and exposure to antimicrobials and neonatal intensive care unit colonization. Most LOS causative organisms often translocate from the gut (predominantly Enterobacteriaceae) [1,2], respiratory tract (predominantly *Acinetobacter*) [3], and skin (predominantly *Staphylococcus*) [4,5]. However, the causative microorganisms of NEC and LOS are not consistently associated with the gut microbiome [2].

Although the microbes involved are non-specific, gut microbial dysbiosis may result in NEC, LOS, bronchopulmonary dysplasia (BPD), and retinopathy of prematurity [6,7]. The pathogenesis of NEC is multifactorial and not well understood [8]. In a previous study, the abundances of Bacteroides and Actinobacteria in infants with NEC at birth were considerably higher than those in the control group. The NEC group had higher abundances of *Alphaproteobacteria*, *Betaproteobacteria*, *Sphingomonas*, and *Lactobacillus*, whereas the control group had significantly higher abundances of *Gammaproteobacteria*, *Enterobacteriaceae*, and *Clostridiaceae* [9].

The gut microbiome contains sepsis-causing pathogenic strains that may increase in abundance before LOS onset. However, only a few previous studies have focused on neonates with LOS, limiting the understanding of the effect of the fecal microbiota in preterm infants (*n* = 19, 13, 7, and 2 neonates [10,11,12,13]) in developed countries (Singapore [12] and the US [11,13]). The 19 LOS-causing isolates are *S. aureus* (*n* = 6); *S. agalactiae*, *Serratia marcescens* (all *n* = 3); *E. faecalis*, *E. coli*, and *K. pneumoniae* (all *n* = 2); and *Enterobacter cloacae* (*n* = 1) [13]. The 13 LOS-causing isolates are *K. pneumoniae*, *S. agalactiae* (*n* = 3), *E. coli*, *S. aureus*, *S. epidermidis* (*n* = 2), and *Proteus mirabilis* (*n* = 1) [12]. The seven LOS-causing isolates are *Staphylococcus aureus* and *S. epidermidis* (both *n* = 2), *Enterococcus faecalis*, *Streptococcus agalactiae*, and *Escherichia coli* (all *n* = 1) [11]. The two LOS-causing isolates are *Klebsiella pneumoniae* [10].

Overall, the association or causation of the fecal microbiome in VLBW infants with severe infections (either LOS or NEC) are poorly understood, with limited studies, particularly in resource-limited settings where severe infection and multidrug resistance are high [14]. NEC and LOS prevention and management require new microbial interventions, such as complementary synbiotics, synergistic symbiotics, and predatory bacteria (as living antibiotics, regardless of pan-drug resistance [15]).

## 2. Materials and Methods

### 2.1. Study Design and Pateint Domains

In this case-control study, we analyzed secondary dataset from a randomized controlled trial (RCT) that examined the effects of oral care with breast milk and sterile water intake in VLBW infants [16]. This study was conducted between July 2018 and June 2020 at the Level IV neonatal intensive care unit of Songklanagarind Hospital, affiliated with Prince of Songkla University, Thailand. This study was approved by the Ethics Committee of the Faculty of Medicine, Prince of Songkla University (REC. 60–455–01–1) and registered in the Thai Clinical Trials Registry (TCTR20180306002). Informed consent was documented before enrollment in the study.

The previous RCT [16] included only VLBW (BW < 1500 g) neonates born in a hospital. Exclusion criteria included maternal death; neonatal contraindications to mother’s own milk; neonates with chromosomal or syndromic anomalies, gut anomalies, moribund by the attending neonatologist; and instances where the parents did not consent to participate. To improve the refinement of the sequence dataset for fecal microbiome analysis from rarefaction curves, we decided on a sequencing depth of 10,000 for further analysis (Supplementary File Figure S1).

### 2.2. Feeding Care

Gastric feeding with the mother’s own milk, preterm formula, or both were initiated a few days after birth in stable neonates via an orogastric tube. Bovine milk-derived fortifiers or concentrated formula milk were used for fortification. No probiotics, prebiotics, or lactoferrin were administered.

### 2.3. Outcome and Definitions

The primary outcome was the biodiversity and abundance of the fecal microbiome in VLBW neonates with severe infection (sInfection group, either NEC or LOS) and non-severe infection (non-sInfection group, neither NEC nor LOS).

NEC was diagnosed considering Bell’s criteria stage II–III [17]. Clinical sepsis is diagnosed by recognizing a combination of clinical signs of infection and antibiotic therapy for at least 3 days [18,19]. LOS was defined as the culture-proven sepsis (clinical sepsis with at least one blood culture of Gram-negative bacteria, at least two blood cultures of commensal organisms, or candidemia) after 72 h of postnatal age. At least 1 mL of blood was processed in an automatic blood culture machine (BacT/Alert™ [bioMérieux, Marcy-l’Étoile, France] or BACTEC FX™ [BD Biosciences, Franklin Lakes, NJ, USA]).

Premature rupture of membranes (PROM) was defined as >18 h before delivery. Maternal antibiotic and antenatal steroid use were defined as maternal antimicrobial treatment within 7 days leading up to delivery and any dose of maternal dexamethasone used, respectively. Patent ductus arteriosus was diagnosed based on clinical and echocardiographic criteria. Moderate to severe BPD was diagnosed by treatment with oxygen for ≥28 days until 36 weeks postmenstrual age (gestational age plus postnatal age) or discharge [20].

The duration of antibiotic use was defined as the cumulative day of antimicrobial use until the date of a fecal collection. The time to fully enteral feeding was defined as the duration taken by VLBW neonates to tolerate 120 mL/kg/day of enteral feed. Oral care using mother’s milk was presented by the number of neonates who randomly received oropharyngeal therapy via own mother’s milk divided by the total number of neonates who received oropharyngeal therapy of either the own mother’s milk or sterile water [16]. The percentage volume of enteral breast milk was analyzed by the proportion of human milk volume divided by the total volumes of both human and formula milk.

### 2.4. Fecal Samples for DNA Extraction

Fecal samples (at least 0.5 g) were immediately collected by registered nurses from the diapers of each neonate at 28 days of age into a microcentrifuge tube (Eppendorf, Hamburg, Germany). Fecal samples were stored at −20 °C until DNA extraction.

DNA was extracted using Zymobiomics (QIAamp Fast DNA Mini Kit; Qiagen, Hilden, Germany) [21]. To monitor potential contamination, reagent-only controls were included in the extraction process. These controls comprised all reagents used in sample collection, DNA/RNA preservation, library preparation, and sequencing but did not contain added DNA. This step was crucial for detecting contamination originating from the reagents or laboratory environment. We assessed the quality and quantity of the extracted DNA by a NanoDrop spectrophotometer and 1.2% agarose gel electrophoresis.

Amplicon sequencing was performed targeting the V3-V4 regions of the 16S rRNA gene using an Illumina MiSeq (San Diego, CA, USA). Microbiota analysis was conducted using the QIIME 2 2021.11 software package [22]. The DADA2 plugin within QIIME 2 was employed to facilitate quality filtering, denoising of the sequence data, and assignment of operational taxonomic units (OTUs). Taxonomic classification of the OTUs was performed using the SILVA ribosomal RNA database based on a 97% similarity threshold, allowing for accurate representation of microbial diversity. The confidence level for species identification was determined using bootstrap values, maintaining a threshold of 70%. Additionally, sequences classified as Archaea, Eukaryotes, and mitochondria sequences were excluded from the analysis using the microDecon package (version 1.0.2) in R software (version 4.3.3; The R Foundation for Statistical Computing, Vienna, Austria).

### 2.5. Statistical Analysis

R software (version 4.3.3) was used to analyze the baseline characteristics between the sInfection and non-sInfection groups by chi-squared or Fisher’s exact test (for categorical variables), or *t*-test or rank sum test (for normally or non-normally distributed continuous variables, respectively).

Bioinformatic and statistical analyses of microbiome data were conducted by QIIME 2 2021.11 [22]. Alpha-diversity was represented by Shannon’s diversity index and observed using OTUs, Pielou’s evenness, and Faith’s phylogenetic diversity. The *p*-value of alpha diversity was adjusted based on the baseline characteristics with *p* < 0.2 (Table 1). Beta diversity was represented by unweighted and weighted UniFrac distances. The *q*-value of beta diversity was corrected by false discovery rate. Relative abundance of taxa was represented as percentages, and differential abundance of taxa was compared by linear discriminant analysis (LDA) effect size (LEfSe) [21]. In the analysis of the relative abundance of different taxa, the main gut microbiota was described by the percentage of taxa >1% in either the sInfection or non-sInfection groups. The LEfSe method was used to evaluate significant differences in operational taxonomic units between the sInfection and non-sInfection groups. The threshold of LEfSe analysis for a discriminatory feature of the LDA score [log10] was set at 4.0.

## 3. Results

Sixty-three participants were randomly enrolled; 12 of these neonates were excluded from analysis based on sequencing depth < 10,000 in rarefaction curves. Thus, we finally analyzed 51 neonates with a mean BW and gestational age (GA) of 1035.8 ± 267.3 g and 28.6 ± 2.2 weeks, respectively, to determine clinical outcomes and evaluate gut microbiomes. The primary characteristics of the sInfection (18%, 9/51) and non-sInfection (82%, 42/51) groups are presented in Table 1. The sInfection group contained 5 and 4 neonates with NEC and LOS, respectively. The NEC episodes for each infant were diagnosed on days 8, 16, 25, and 28 of postnatal age. The LOS episodes for each infant were diagnosed on days 3, 6, 10, 20, and 28 of postnatal age. Pathogenic isolates from neonates with LOS included *Acinetobacter baumannii*, *E. cloacae*, *K. pneumoniae*, and *S. epidermidis*. The sInfection group had more cases of moderate-to-severe BPD and longer durations of invasive ventilation, non-invasive ventilation, antimicrobial use, and a longer time to achieve full enteral feeding than did the non-sInfection group (Table 1).

Univariate analysis shows no significant differences in the alpha and beta diversities between the sInfection and non-sInfection groups (Table 2). Multivariate analysis revealed that alpha (before and after adjusting for GA, BW, PROM, moderate-to-severe BPD, percentage volume of enteral breast milk, duration of invasive ventilation, duration of non-invasive ventilation, duration of antibiotic use, and date of full enteral feeding) and beta (before and after correcting for false discovery rate) diversities between the sInfection and non-sInfection groups were not significantly different.

**Figure 1:**
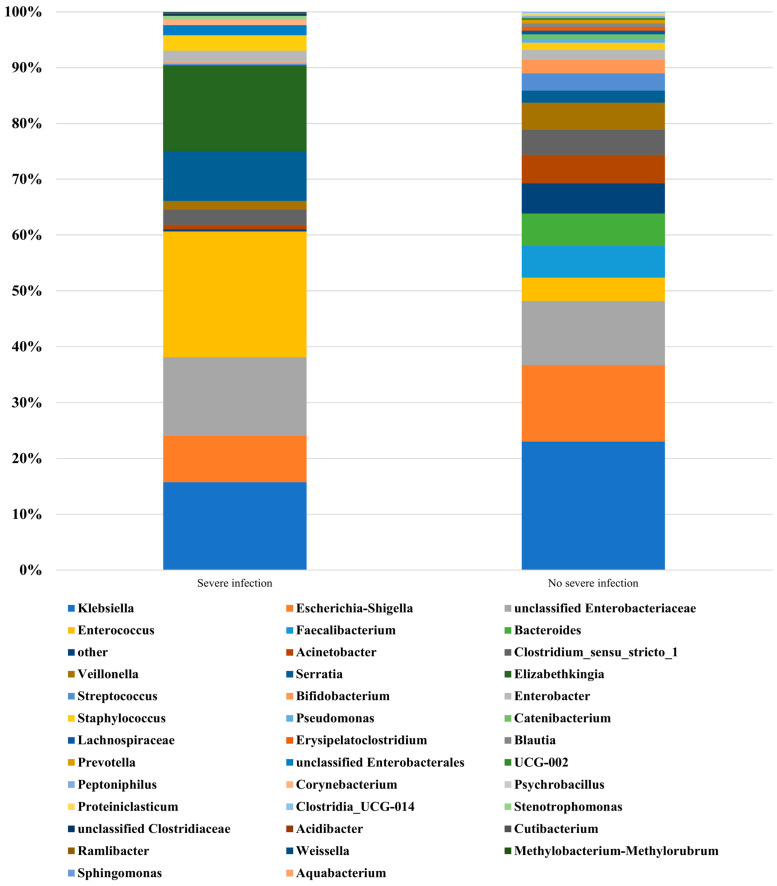
Relative abundance of isolated genera from fecal samples in each group of very low birth weight infants with and without severe infection (severe infection and no severe infection, respectively).

The main gut microbiome in the sInfection and non-sInfection groups are presented in Table 3. A summary of the top 31 genera is presented in Figure 1. Among the main gut bacteria (summary of genera in both groups, >1%), Faecalibacterium and Bacteroides were found only in the non-sInfection group. Based on a pooled mean difference of >1% at the genus level, Enterococcus (18.3%), Elizabethkingia (15.3%), Serratia (6.8%), unclassified Enterobacteriaceae (2.6%), unclassified Enterobacterales (1.8%), Staphylococcus (1.5%), and Corynebacterium (1.0%) had a higher relative abundance in the sInfection group, whereas Klebsiella (7.3%), Faecalibacterium (5.8%), Bacteroides (5.8%), Escherichia-Shigella (5.3%), Acinetobacter (4.3%), Veillonella (3.3%), Streptococcus (2.8%), Bifidobacterium (2.2%), and Clostridium_sensu_stricto_1 (1.8%) had a higher abundance in the non-sInfection group. Based on a pooled mean difference of >0.5% at the species level, Bacteroides fragilis (4.1%) and Clostridium butyricum (1.8%) were found only in the non-sInfection group, whereas neither isolate was found in the sInfection group.

Analysis of four neonates with LOS showed that the relative abundance of pathogenic organisms (top three genera or pathogenic isolates from gut microbiota of each neonate) were *A. baumannii* (*Escherichia-Shigella* 43.9%, *Enterococcus* 41.5%, *Klebsiella* 11.0%, neither *Acinetobacter* nor *A. baumannii*), *E. cloacae* (*Serratia* 47.2%, *Enterobacter* 10.4%, *Klebsiella* 9.9%), *K. pneumoniae* (*Enterococcus* 93.3%, *Stenotrophomonas* 3.3%, *Klebsiella* 0.6%), and *S. epidermidis* (*Clostridium_sensu_stricto_1* 30.4%, *Escherichia-Shigella* 24.6%, *Klebsiella* 22.7%, neither *Staphylococcus* nor *S. epidermidis*).

**Figure 2:**
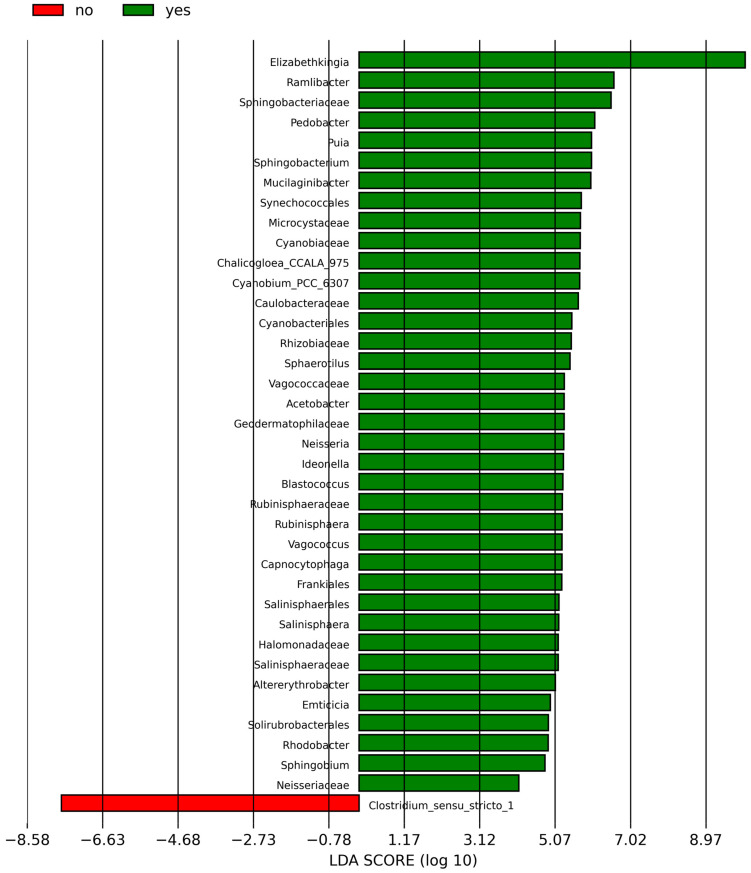
Linear discriminant analysis effect size (LEfSe) indicating differentially abundant microbial clades; the red (no; non-severe infection) and green (yes; severe infection) bar charts represent the significantly abundant taxa in cases without and with severe infection, respectively.

**Figure 3:**
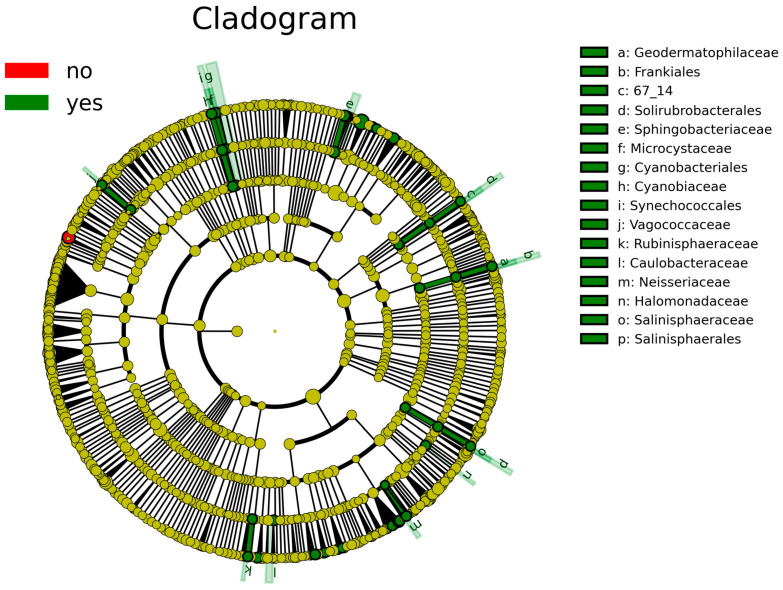
Linear discriminant analysis effect size (LEfSe) indicating differential abundance of microbial clades: Cladogram illustrates significantly abundant clades (red [no] and green [yes] circles represent the non-severe infection and severe infection groups, respectively). The dark yellow circles represent non-significant microbial clades between groups. The green bars [a–p] did not include the genus and species levels.

According to the analysis of the differential taxa abundance, LEfSe analysis using an LDA score >4 identified only one key taxon (genus *Clostridium_sensu_stricto_1*) in the non-sInfection group, which was significantly more abundant than in the sInfection group (Figure 2), whereas 37 taxa in the sInfection group (*Elizabethkingia*, *Ramlibacter*, *Sphingobacteriaceae*, *Pedobacter*, *Puia*, *Mucilaginibacter*, *Sphingobacterium*, *Synechococcales*, *Cyanobiaceae*, *Cyanobium_PCC_6307*, *Acetobacter*, *Microcystaceae*, *Chalicogloea_CCALA_975*, *Caulobacteraceae*, *Cyanobacteriales*, *Rhizobiaceae*, *Sphaerotilus*, *Vagococcaceae*, *Vagococcus*, *Ideonella*, *Frankiales*, *Geodermatophilaceae*, *Blastococcus*, *Neisseria*, *Capnocytophaga*, *Rubinisphaeraceae*, *Rubinisphaera*, *Halomonadaceae*, *Salinisphaerales*, *Salinisphaeraceae*, *Salinisphaera*, *Altererythrobacter*, *Emticicia*, *Solirubrobacterales*, *Rhodobacter*, *Sphingobium*, and *Neisseriaceae*) were significantly more abundant than in the non-sInfection group. The red and green circles in the cladogram represent significantly abundant clades in the non-sInfection and sInfection groups, respectively, and green bars in the sInfection group did not include the genus and species levels) (Figure 3).

## 4. Discussion

The alpha (unadjusted and adjusted models by clinical factors) and beta (unadjusted and adjusted models by false discovery rate) diversities did not significantly differ between the non-sInfection and sInfection groups. The main gut bacteria were found in only some taxa in the non-sInfection group (1% mean difference at the genus level [*Faecalibacterium* and *Bacteroides*] and >0.5% mean difference at the species level [*B. fragilis* and *C. butyricum*]). Significant mean differences were observed between the top three highest relative abundances at the genus level between the non-sInfection group (*Klebsiella*, *Faecalibacterium*, and *Bacteroides*) and sInfection group (*Enterococcus*, *Elizabethkingia*, and *Serratia*). *Clostridium_sensu_stricto_1* was significantly more abundant in the non-sInfection group than in the sInfection group.

The main taxa in the fecal microbiota differed across age groups. Proteobacteria, Actinobacteria, and Firmicutes were previously shown to be the most abundant phyla in the guts of neonates [23,24], infants [24], and children (until adulthood) [25], respectively. In the present study, the most abundant microorganisms in the gut microbiota of VLBW infants were Proteobacteria, Gammaproteobacteria, and *Klebsiella*. *Klebsiella* spp. are enriched in the fecal microbiome of preterm infants and are the most common pathogens causing LOS in developing countries [1].

The main gut bacteria at the genus level, *Faecalibacterium* and *Bacteroides,* were observed only in the non-sInfection group. *Faecalibacterium* are butyrate producers. Butyrate is defined as a postbiotic and may maintain the integrity of the gut lining [26]. The main gut bacteria at the species level, *B. fragilis* and *C. butyricum*, were found in the non-sInfection group. First, *B. fragilis*, an obligatory anaerobic Gram-negative bacillus, colonizes the normal microbiota of a healthy human colon. Polysaccharide A produced by *B. fragilis* is involved in host-microbiota symbiotic relationships and plays an immunomodulatory role by inducing regulatory T cells and suppressing pro-inflammatory T helper 17 cells [27]. *Clostridium butyricum* is an anaerobic, endospore-forming, Gram-positive, butyric acid-producing bacillus. It is used as a probiotic in Japan (e.g., for the prophylaxis and therapy of antibiotic-associated diarrhea in children [28] and hematopoietic stem-cell transplantation [29]); however, only 5 (0.08%) of 6576 cases of *C. butyricum* bacteremia were associated with probiotic use in Japan [30].

Only *Clostridium_sensu_stricto_1* was more abundant in the non-sInfection group than in the sInfection group. *Clostridium_sensu_stricto_1*, which produces butyrate, is an important anaerobic bacterium found in the human gut. In a previous RCT, 106 healthy adults were enrolled to receive either synbiotics (containing *Lactobacillus rhamnosus*, *Bifidobacterium lactis*, and fructooligosaccharide) or a placebo for 8 weeks. Synbiotic supplementation enriched beneficial bacteria (*Clostridium_sensu_stricto_1*, *Lactobacillus*, and *Bifidobacterium*) and several functional pathways related to amino acid and short-chain fatty acids biosynthesis [31]. In preterm infants, the *Clostridium_sensu_stricto_1* in gut microbiota was higher in the breast milk group (>70% human milk intake) than in the preterm formula group (<70% human milk intake) 3 weeks after birth (*p* < 0.05) [32].

However, this study had some limitations. First, the sample size was small in both the case and control groups. Although some beneficial bacteria were found in the non-sInfection group, they were not found in any samples in the non-sInfection group. Further studies are needed to identify the causative and protective factors. However, these new findings may provide multimodal strategies for infection prevention and control, particularly for probiotics and postbiotics. Second, all fecal samples were collected on day 28 following the neonatal period (defined as postnatal age less than 28 days) and not on the date of severe infection. A correlation was observed between the gut microbiota and severe infections; however, causation analysis and sequencing did not clarify these results. Further studies are needed to evaluate the dynamic changes in gut microbiota over time in VLBW infants. Third, the enteral breast milk volume in the non-sInfection group was higher than that in the sInfection group, but the difference was not significant. This percentage was only considered for infants subjected to tube and bottle feeding, did not include breastfeeding, and was adjusted for biodiversity. Finally, we only focused on taxonomic differences and did not perform functional pathway analyses (e.g., metagenomics, mechanistic pathways of butyrate producers, or infection resistance) to directly link the microbial composition with butyrate production and its protective effects.

## 5. Conclusions

Microbial biodiversity, including adjusted alpha and corrected beta diversity, in the fecal microbiome did not significantly differ between the sInfection (NEC or LOS) and non-sInfection groups. The main gut microbiota were found only in the non-sInfection group, particularly butyrate- (*C. butyricum* and *Faecalibacterium*) and polysaccharide A (*B. fragilis*)-producing taxa. *Clostridium_sensu_stricto_1* was more abundant in the non-sInfection group than in the sInfection group. A microbiome-targeting treatment may allow for tailored early interventions and targeted treatments. Further studies are required to explore both synbiotic and symbiotic (for example, probiotics and butyrate producers) therapeutics as a potential therapeutic option for NEC or LOS in VLBW infants.

## Figures and Tables

**Table 1:** Primary characteristics of cases with and without severe infection.

Baseline Characteristics	sInfection (*n* = 9)	Non-sInfection (*n* = 42)	*p*-Value
Gestational age, weeks *	27.3 ± 1.9	28.9 ± 2.2	0.06
Birth weight, g *	882 ± 229	1069 ± 266	0.06
Male, *n* (%)	3 (33.3)	25 (59.5)	0.27
Appropriate for gestational age, *n* (%)	8 (88.9)	32 (76.2)	0.66
Cesarean delivery, *n* (%)	7 (77.8)	29 (69.0)	0.71
Premature rupture of membranes, *n* (%)	2 (22.2)	7 (16.7)	0.09
Maternal antibiotics, *n* (%)	2 (22.2)	11 (26.2)	>0.99
Antenatal steroid, *n* (%)	7 (77.8)	27 (64.3)	0.70
1 min Apgar score ^†^	5 (4–6)	6 (3–8)	0.28
5 min Apgar score ^†^	8 (5–8)	8 (7–9)	0.27
Number of endotracheal intubations, *n* (%)	8 (88.9)	30 (71.4)	0.42
Duration of invasive ventilation, days ^†^	5 (2–11)	1 (0–3)	0.01
Duration of non-invasive ventilation, days ^†^	38 (28–51)	11 (7–36)	0.02
Respiratory distress syndrome, *n* (%)	6 (66.7)	36 (85.7)	0.19
Surfactant administration, *n* (%)	5 (55.6)	17 (40.5)	0.47
Patent ductus arteriosus, *n* (%)	4 (44.4)	13 (31.0)	0.46
Moderate to severe bronchopulmonary dysplasia, *n* (%)	4 (44.4)	5 (11.9)	0.04
Duration of antibiotic use, days ^†^	23 (18–26)	6 (3–12)	0.001
Date of full enteral feeding, days ^†^	27 (23–32)	12 (7–17)	<0.001
Date of regained birth weight, days *	9.0 ± 4.1	8.3 ± 3.6	0.60
Oral care by mother’s own milk, *n* (%)	5 (55.6)	20 (47.6)	0.73
Percentage volume of enteral breast milk ^†^	30 (4–94)	93 (68–99)	0.08

* The data are presented as the mean ± standard deviation. ^†^ The data are shown as the median (interquartile range).

**Table 2:** Fecal microbiome biodiversity in cases with and without severe infection.

Alpha Diversity	sInfection (*n* = 9)	Non-sInfection (*n* = 42)	*p*-Value	*p*-Value *
Shannon diversity ^a^	3.2 (2.4–3.7)	3.4 (3.1–4.2)	0.19	0.28
Observed using operational taxonomic units ^a^	34 (31–42)	49 (39–161)	0.06	0.74
Faith’s phylogenetic diversity ^a^	9.0 (7.5–12.7)	8.4 (7.6–30.6)	0.49	0.59
Pielou’s evenness ^b^	0.6 ± 0.1	0.6 ± 0.1	0.50	0.33
Beta diversity	Permutations	pseudo-F	*p*-Value	*q*-Value **
Unweighted UniFrac distance	999	1.06	0.29	0.29
Weighted UniFrac distance	999	1.81	0.08	0.08

^a^ Median (interquartile range). ^b^ Mean ± standard deviation. * *p*-Value was adjusted for gestational age, birth weight, premature rupture of membranes, moderate-to-severe bronchopulmonary dysplasia, percentage volume of enteral breast milk, duration of invasive ventilation, duration of non-invasive ventilation, duration of antibiotic use, and date of full enteral feeding. ** *q*-Value was corrected using the false discovery rate.

**Table 3:** Relative abundance from gut microbiota in cases with and without severe infection.

Taxa	Bacteria	sInfection (*n* = 9)	Non-sInfection (*n* = 42)
Phyla	Proteobacteria	52.9%	59.2%
	Firmicutes	30.2%	30.9%
	Bacteroidota	15.5%	6.8%
	Actinobacteriota	1.4%	2.6%
Classes	Gammaproteobacteria	52.8%	58.8%
	Bacilli	25.6%	10.5%
	Bacteroidia	15.5%	6.8%
	Clostridia	3.1%	14.9%
	Negativicutes	1.6%	5.1%
	Actinobacteria	1.4%	2.6%
Genera	*Klebsiella*	15.8%	23.0%
	*Escherichia-Shigella*	8.3%	13.7%
	*unclassified Enterobacteriaceae*	14.1%	11.5%
	*Enterococcus*	22.5%	4.1%
	*Faecalibacterium*	0%	5.8%
	*Bacteroides*	0%	5.8%
	*Acinetobacter*	0.8%	5.1%
	*Clostridium_sensu_stricto_1*	2.7%	4.5%
	*Veillonella*	1.6%	4.8%
	*Serratia*	9.0%	2.2%
	*Elizabethkingia*	15.3%	0%
	*Streptococcus*	0.3%	3.1%
	*Bifidobacterium*	0.3%	2.4%
	*Enterobacter*	2.1%	1.8%
	*Staphylococcus*	2.7%	1.2%

## Data Availability

The raw data supporting the conclusions of this study will be provided available by the authors on request. The data are not publicly available due to privacy.

## Supplementary Materials

The following supporting information can be downloaded at https://www.mdpi.com/article/10.3390/nu17081329/s1, Figure S1: Rarefaction curves demonstrate the sampling depth at 10,000 sequences.
