# Supplementary figures and images for: Butyrate Producers in Very Low Birth Weight Infants with Neither Culture-Proven Sepsis nor Necrotizing Enterocolitis
Source: Nutrients. 2025 Apr 11;17(8):1329. doi: 10.3390/nu17081329 (PMC12029688; doi:10.3390/nu17081329)

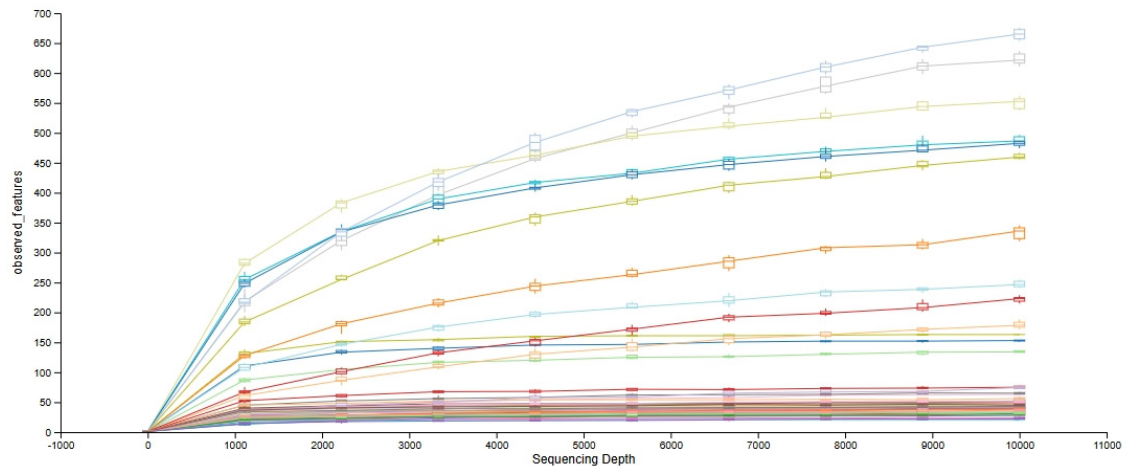

**Figure S1.** Rarefaction curves demonstrate the sampling depth at 10,000 sequences.

Supplement: Supplementary file 1 [file nutrients-17-01329-s001.zip › nutrients-3413560-supplementary.pdf]
